# New eurymeline leafhoppers (Hemiptera, Cicadellidae, Eurymelinae) from Eocene Baltic amber with notes on other fossil Cicadellidae

**DOI:** 10.3897/zookeys.726.21976

**Published:** 2018-01-10

**Authors:** Christopher H. Dietrich, M. Jared Thomas

**Affiliations:** 1 Illinois Natural History Survey, Prairie Research Institute, University of Illinois, 1816 S. Oak St., Champaign, IL 61820, USA

**Keywords:** Auchenorrhyncha, Idiocerini, Macropsini, morphology, Phlogisini, Signoretiinae

## Abstract

Two new extinct fossil cicadellid taxa from Eocene Baltic amber, representing the subfamily Eurymelinae (*sensu lato*), are described and illustrated, and their relationships to modern leafhoppers are discussed. *Eoidiocerus emarginatus* **gen.** and **sp. n.** is the oldest known representative of the tribe Idiocerini. The new genus resembles some modern Afrotropical and Indomalayan idiocerine genera but differs in having the gena relatively narrow. *Archipedionis obscurus* **gen.** and **sp. n.**, is the first well-preserved fossil representative of Macropsini to be described in detail. Previous reports of this tribe from Baltic amber, while credible, included too little morphological information to assess their relationships. Additional comparative notes are provided for previously described fossil taxa belonging to Idiocerini and Macropsini from the Oligocene of Germany. The new combinations *Oncopsis sepultus sepultus* (Statz, 1950), **comb. n.** and *Oncopsis sepultus austerus* (Statz, 1950), **comb. n.** are proposed for taxa previously included in *Bythoscopus* Germar. The previously unplaced cicadellid fossil taxon *Priscacutius denticulatus* Poinar & Brown, 2018 from mid-Cretaceous Myanmar amber is newly placed in subfamily Signoretiinae, tribe Phlogisini, and represents the oldest known member of this subfamily, the only one known from the fossil record and only the second modern cicadellid subfamily confirmed by direct fossil evidence to have been present during the Cretaceous period.

## Introduction

The fossil record of leafhoppers (Cicadellidae), a group of sap-sucking hemipteran insects comprising >20,000 described extant species worldwide, is poorly documented, with fewer than 100 confirmed fossil species having been formally described so far from the Cretaceous (Oman 1937, Shcherbakov 1986, Hamilton 1990, 1992, Poinar and Brown 2018), Eocene (reviewed by Szwedo 2002, 2005, Dietrich and Gonçalves 2014, Gröhn 2016) and Oligo-Miocene (Statz 1950, Dietrich and Vega 1995). Several additional fossils listed as belonging to “Cicadelloidea” by Metcalf and Wade (1966) have either been transferred to other families or require further confirmation.

Recent molecular phylogenetic studies of leafhoppers have attempted to estimate the ages of various cicadellid lineages (Krishnankutty 2012, Catanach 2013, Krishnankutty et al. 2016, Wang et al. 2016, Dietrich et al. 2017) but have been hindered by the paucity of well-preserved fossil leafhoppers available for calibrating the ages of nodes on phylogenies. Although molecular divergence time estimates consistently suggest that many modern cicadellid subfamilies originated during the Cretaceous, only one such subfamily, Ledrinae, has been reported previously from Cretaceous-age fossil material (Hamilton 1990, Shcherbakov 1992).

Six modern cicadellid subfamilies (Aphrodinae, Bathysmatophorinae, Eurymelinae, Megophthalminae, Mileewinae and Typhlocybinae) have their oldest representatives recorded from Eocene Baltic amber (Dietrich and Gonçalves 2014). A record from Baltic amber of subfamily Coelidiinae (based on “*Jassus*” *immersus* Germar and Berendt, 1856), considered to “very probably” represent this subfamily (Szwedo and Sontag 2009), requires further verification. Records of Deltocephalinae (as “Deltocephalinae cf. Paralinnini”) and Neocoelidiinae (a nymph, as “Neocoelidinae”) based on photographs by Gröhn (2016) also require further study and confirmation.

Judging from the numerous amber specimens offered for sale online over the past few years by dealers in Lithuania, Poland and elsewhere in Europe, most of the leafhoppers preserved in Baltic amber are nymphs, and many of these are difficult to place taxonomically and phylogenetically, given the still highly incomplete knowledge of the morphology of modern leafhopper nymphs (reviewed by Dmitriev 2002). One early-instar nymph was placed by Szwedo and Gebicki (2002) in its own subfamily, Nastlopiinae, but this placement cannot, at present, be thoroughly evaluated because the early (first and second) instar nymphs of most extant leafhopper subfamilies have never been described in the literature and very few specimens are preserved in collections.

The new fossil taxa described herein include the oldest known representative of the leafhopper tribe Idiocerini and the first report of the tribe from fossil amber. Idiocerini, at present, are distributed worldwide with 105 genera and ~700 known extant species. Previous records of fossil Idiocerini consist of three rock fossils from the Oligocene of Germany (Statz 1950, see comparative notes below). Also described below is a new fossil representative of Macropsini, a tribe previously recorded from Baltic amber (as “Macropsinae”, Szwedo 2002) but based on specimens very incompletely described and illustrated by Germar and Berendt (1856).

## Material and methods

Fossil specimens were obtained from amber dealers in Palanga, Lithuania. Morphological characters were assessed by examination of the specimens using an Olympus SZX-12 dissecting microscope. Specimens were prepared by grinding flat facets in strategic locations to obtain a clear field of view for detailed photomicrographs according to Nascimbene and Silverstein (2000) and Bisulca et. al. (2012). Photomicrographs were taken using a Zeiss SteREO Discovery V20 zoom stereomicroscope with a Plan-Apochromat S 0.63x f/Reo WD=81 mm objective. Drawings were prepared either with a camera lucida or by tracing over photographs of the specimens. For bilaterally symmetrical parts of the head and thorax obscured by fractures and other flaws in the amber, drawings (Fig. 2A, B, F, G) were prepared by tracing one half (the fully visible side) with the camera lucida and reconstructing the other half using its mirror image. All specimens examined are deposited in the Paleontological Collection of the Illinois Natural History Survey, Champaign, Illinois, USA. Morphological terminology follows Dietrich (2005).

## Taxonomy

### Subfamily Eurymelinae

Taxon classificationAnimaliaHemipteraCicadellidae

Amyot & Serville, 1843

#### Note.

The concept of Eurymelinae adopted here is narrower than that of Hamilton (1983) but broader than those of Oman et al. (1990) and Dietrich (2005). It includes Eurymelinae, Idiocerinae and Macropsinae, sensu Oman et al. (1990) and the latter two taxa are treated as tribes of Eurymelinae (following Hamilton 1983). A recent large-scale molecular phylogenetic analysis of Membracoidea (Dietrich et al. 2017) placed Idiocerini and Macropsini within a well-supported monophyletic group also including Eurymelinae, *sensu* Oman et al. (1990).

### Tribe Idiocerini Baker, 1915

#### Eoidiocerus gen. n.

Taxon classificationAnimaliaHemipteraCicadellidae

http://zoobank.org/690668B2-F892-4941-BD6E-BC715DE087CE

##### Type species.

*Eoidiocerus emarginatus* sp. n.; by present designation and monotypy.

##### Diagnosis.

This genus differs from other described genera of Idiocerini in having the following combination of traits: head with fine arcuate striations above ocelli, ocelli situated above mid-height of eye, gena strongly emarginate below eye; hind femur macrosetal formula 2+1; female abdominal sternite VII strongly emarginate, exposing base of ovipositor; length of ovipositor more than half that of entire abdomen.

##### Description.

Head in dorsal view with crown slightly shorter medially than next to eyes; face slightly longer than width across eyes, texture shagreen, area of vertex above ocelli with inconspicuous, fine arcuate parallel striations; ocelli approximately equidistant between eyes and midline, situated above mid-height of eyes; lateral frontal suture nearly straight, extended from antennal pit to ocellus; antennal ledge carinate but only weakly produced over antennal base; antenna shorter than head width, arista attenuate, with conspicuous preapical seta extended mesad; gena strongly concave and narrow below eye, partly exposing small proepisternum; lorum convex, extended nearly to lateral margin of face; anteclypeus broadened near apex; rostrum extended slightly past middle coxae, distal segment somewhat expanded toward apex. Pronotum shagreen, with indistinct transverse rugae. Forewing elongate, appendix broad, extended to wing apex, bordering first and second apical cells; vein R with three branches extended to wing margin; crossvein s absent; with two r-m and three m-cu crossveins (two closed subapical cells); vein CuA reaching submarginal vein near midlength of appendix; claval veins distinct. Front femur with AM1 strongly reduced; intercalary row with several long, fine setae; tibia cylindrical, with conspicuous setae only at apex. Middle femur and tibia without macrosetae. Hind femur macrosetal formula 2+1; tibia strongly flattened, distance between dorsal setal rows much less than distance between dorsal and ventral rows, row AD with fewer macrosetae than PD, row AV macrosetae distributed along distal 3/4 of tibia, row PV with alternating short and long tapered setae through most of length, tarsomere I with dorsoapical pair of macrosetae well developed, without plantar setae, pecten with 2 platellae. Female pygofer and ovipositor narrow and elongate, occupying 3/4 total length of abdomen; sternite VII with deep median parabolic emargination, exposing base of ovipositor.

##### Etymology.

The genus name, a masculine noun, combines the Greek word *eos* (dawn) with *Idiocerus*, the name of the type genus of Idiocerini, referring to the status of the fossil as the oldest known representative of Idiocerini..

##### Notes.

Placement of *Eoidiocerus* in Idiocerini is unequivocal and supported by the presence of several synapomorphic features diagnostic for that tribe, including: head broader than pronotum, crown short, ocelli on face distant from dorsal margin and well separated from eyes, lateral frontal sutures present and extended to ocelli; pronotum in dorsal view with anterior margin not extended anteriad of eyes; chaetotaxy of front and middle legs strongly reduced; forewing appendix broad and extended to wing apex. *Eoidiocerus* resembles several modern idiocerines in most external structural features. Its most distinctive diagnostic traits are the arcuate series of fine striations on the vertex above the ocelli, present in several modern genera (e.g., *Idiocerus* Lewis, 1834, *Idioceroides* Matsumura, 1912; see also Webb 1983b), the relatively long and narrow face (occurring also in some Paleotropical genera, e.g., *Chunra* Distant, 1908), the distinctly emarginate, relatively narrow gena (broad and not, or very weakly, emarginate in most modern idiocerines), and the greatly elongated female pygofer and ovipositor, which occurs also in some modern species of *Idiocerus*. The forewing venation of the only available specimen is poorly delimited but the visible parts suggest that the venational pattern in this genus is similar to that exhibited by most modern genera of the tribe, i.e., only two closed anteapical cells are present and the appendix borders only two apical cells rather than three as in some genera from South Asia and Madagascar (Viraktamath 2007, Krishnankutty and Dietrich 2011). In the structure and proportions of the head, pronotum and mesonotum, the new genus is perhaps most similar to *Cafixia* Webb, 1983b, a genus represented by a single species occurring in South Africa, but *Eoidiocerus* differs in having the gena distinctly emarginate below the eye and exposing the small, flaplike proepisternum. Modern idiocerine genera known to have the gena distinctly emarginate below the eyes are *Idioceroides* from East Asia, and *Tumocerus* Evans, 1941 and *Quilopsus* Webb, 1983a from western Australia. *Idioceroides* differs in having the ocelli relatively high and laterad on the face and the lateral frontal sutures reduced. *Tumocerus* and *Quilopsus* differ in numerous respects, including having the face much wider than long, the lateral frontal sutures nearly vertical in orientation, and the portion of the vertex dorsad of the ocelli relatively short and lacking arcuate striations (Webb 1983a: figs 449, 464). The emarginate gena of *Eoidiocerus* also approaches the condition found in Macropsini, but in that tribe the gena is even more strongly emarginate, the proepisternum is enlarged and the lateral frontal sutures are poorly delimited or absent and not extended to the ocelli.

Previously reported fossil Idiocerini include *Oligoidiocerus pronotumnalis* Statz, 1950, *Idiocerus goeckii* Statz, 1950 and an additional unnamed “*Idiocerus* ?” species from the Oligocene of Germany (Statz 1950). *Oligoidiocerus* apparently lacks an appendix in the forewing (Statz 1950: fig. 17) and, therefore, probably does not belong to this tribe. Its forewing venation is consistent with that of tribe Macropsini but other characters that could confirm its placement in that tribe do not appear to be visible on the fossil (Statz 1950: fig. 58). *Idiocerus goeckii* has the forewing venation well preserved and resembling that of modern species of *Idiocerus* (Statz 1950: fig. 18), differing from *Eoidiocerus* in the apparent lack of vein R1 and crossvein m-cu2. According to the photograph provided by Statz (1950: fig. 59) the specimen he identified as “*Idiocerus* ? sp.” is too poorly preserved to confirm its placement in Idiocerini.

#### Eoidiocerus emarginatus sp. n.

Taxon classificationAnimaliaHemipteraCicadellidae

http://zoobank.org/FAFF0AC4-8C8F-441E-860E-9E8D5581ECE0

Figs 1A–B , 2A–E

##### Description.

Measurements (mm): body length including wings 4.8; head width across eyes 1.4; height of face (crown apex to anteclypeus apex) 1.5; forewing length 3.8; forewing maximum width (across approximately midlength) 1.1 mm; front tibia length 0.7; hind tibia length 1.7; hind tarsus length 0.7; ovipositor length (portion exposed posterad of sternite VII) 1.3. Hind tibia rows PD, AD and AV with 10, 9 and 11 macrosetae, respectively. Other structural features as described for genus. Body apparently uniformly pale brown, without discernible markings or pattern.

##### Etymology.

The species name refers to the emarginate gena.

##### Material examined.

Holotype female, Eocene Baltic amber (37–44 Ma), purchased by the first author from an amber dealer in Palanga, Lithuania. Deposited in the Paleontological Collection of the Illinois Natural History Survey (INHSP 10320).

The exoskeleton of the holotype is well preserved and intact except the femoro-tibial joints and adjacent parts of the left legs have been sheared off, apparently during initial processing of the amber piece, and are missing; most of the tibia and the entire tarsus of the left middle leg are also missing. Variable preservation of different parts of the integument give the impression that the holotype specimen has a pattern of dark markings but these appear to be artifacts.

**Figure 1.**
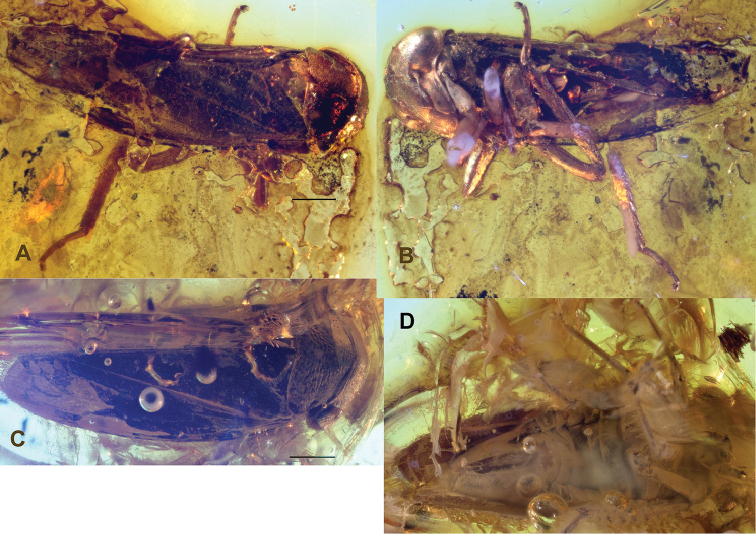
**A–B**, *Eoidiocerus emarginatus* gen. and sp. n. holotype, dorsolateral and ventrolateral habitus **C–D** *Archipedionis obscurus* gen. and sp. n. holotype, dorsal and ventral habitus.

### Tribe Macropsini Evans, 1935

#### Archipedionis gen. n.

Taxon classificationAnimaliaHemipteraCicadellidae

http://zoobank.org/9B6D4F13-9484-4861-A8F1-CE80BED0516D

##### Type species.

*Archipedionis obscurus* sp. n.; by present designation and monotypy.

##### Diagnosis.

This genus differs from other Macropsini in having the following combination of traits: crown shorter medially than next to eye; face with epistomal suture visible; ocelli slightly mesad of antennal pits, coronal pits dorsolaterad of ocelli; lorum not fused to frontoclypeus or anteclypeus; rostrum extended beyond middle coxae. Pronotum angulately produced medially but extended only slightly anterad of eyes in dorsal view, irregularly rugose. Forewing outer anteapical cell open, veins without markings.

##### Description.

Head in dorsal view with crown shorter medially than next to eyes; face relatively broad and short, texture minutely and more or less evenly punctate, ocelli slightly closer to eyes than to midline; coronal pits present dorsolaterad of ocelli; epistomal suture visible; gena strongly concave and narrow below eye, exposing flaplike proepisternum; lorum convex, extended nearly to lateral margin of face, not fused to anteclypeus; anteclypeus parallel-sided with apex truncate; rostrum extended past middle coxae, slender. Pronotum shagreen, with irregular transverse rugae. Forewing elongate, appendix narrow, extended around wing apex; most of membrane opaquely sclerotized; veins somewhat obscure, without obvious markings; inner and middle anteapical cells closed, outer anteapical cell open (crossvein s absent); claval veins distinct. Visible portion of hind wing apex with two closed apical cells, anterior branch of R absent. Front femur and tibia without conspicuous setae. Middle femur and tibia without macrosetae. Hind femur macrosetal formula 2+1; tibia strongly flattened, distance between dorsal setal rows much less than distance between dorsal and ventral rows, row AD with 8 preapical macrosetae (PD not visible in fossil), row AV macrosetae extended most of length of tibia, row PV with numerous close-set slender setae subequal in length, tarsomere I with dorsoapical pair of macrosetae well developed, with two rows of plantar setae, pecten with 2 platellae. Female pygofer relatively short, occupying < half total length of abdomen; sternite VII angulately emarginate, covering base of ovipositor.

##### Etymology.

The genus name, a masculine noun, combines the prefix *archi*- derived from the Greek *archaeos*, meaning old, with *Pedionis*, the name of a modern macropsine genus with similar forewing venation.

##### Notes.

This genus has forewing venation resembling that of the modern genus *Pedionis* Hamilton, 1980, i.e., with the s crossvein delimiting an outer anteapical cell absent, but differs in having the structure of the head more plesiomorphic, resembling *Zelopsis* Evans, 1966. Specifically, the face has the epistomal suture visible and arcuate and the anteclypeus is well delimited laterally and basally by sutures. The pronotum is not strongly produced anteromedially, although it still extended slightly anterad of the eyes medially, and the transverse rugae are only slightly arched anterad medially. Unfortunately, because only one female specimen is known, it is not known whether the structure of the lower part of the face is sexually dimorphic in *Archipedionis*, as is usual among modern macropsines. The elongate rostrum of this genus is apparently unusual in the modern macropsine fauna and has been reported only in *Galboa* Distant, 1909 (Seychelles Islands) and *Paragalboa* Yang, Dietrich & Zhang, 2016 (Madagascar), but also occurs in some species of *Pedionis*.

Three previously described fossil species from Baltic amber have been included in Macropsini: *Bythoscopus homousius* Germar & Berendt, 1856, *B. punctatus* Bervoets, 1910, and *Pediopsis minuta* Bervoets, 1910 (Szwedo 2002). Unfortunately, the only known specimens of these species were apparently destroyed during World War II and the original descriptions and illustrations are not sufficiently detailed to facilitate placement or detailed comparison with the species described here. Nevertheless, information provided in the original descriptions appears to indicate that these previously described species are different from the one described here. According Germar and Berendt (1856), *B. homousius* has the outer anteapical cell of the forewing closed distally (open in *Archipedionis*). *Pediopsis minuta* is much smaller (3 mm vs. 4.5 mm) and has the anterior margins of the head and pronotum much more strongly angulate. *B. punctatus* is similar in size and in the shape of the head and pronotum to *Archipedionis obscurus* but the ocelli are closer to the midline of the face and the frontal sutures are not delimited. Collectively, these three species and the new species described below are the oldest representatives of Macropsini known from the fossil record.

Statz (1950) reported another species of Macropsini, *Macropsis pectoralis* Statz, 1950, from the Oligocene of Germany. The photograph of the holotype provided by Statz (1950: fig. 57) indicates that this fossil is correctly placed in Macropsini based on overall size and the form of the head and pronotum (pronotum angulately extended anterad of eyes) but its forewing venation is only partly visible and other traits that would facilitate detailed comparison with modern taxa are not visible. *Bythoscopus sepultus* Statz, 1950 may also be confidently placed in Macropsini based on the hind wing venation (absence of vein R2+3, submarginal vein not extended along costal margin basad of R4+5). The shape of the head and pronotum (Statz 1950: fig. 7) are indistinguishable from those of the modern Holarctic macropsine genus *Oncopsis* Burmeister, 1838; therefore the new combinations *Oncopsis sepultus sepultus* (Statz), comb. n. and *Oncopsis sepultus austerus* (Statz), comb. n. are proposed here. Two additional fossils placed by Statz (1950) in *Bythoscopus* Germar, 1833 (an isogenotypic junior synonym of *Iassus* Fabricius, 1803), *B. lunatus* Statz, 1950 and *B. robustus* Statz, 1950, also appear to belong to Macropsini but their correct generic placements cannot be determined due to the poor condition of the fossils.

#### Archipedionis obscurus sp. n.

Taxon classificationAnimaliaHemipteraCicadellidae

http://zoobank.org/EF78935C-60F2-4F05-83DC-615570BDA0E5

Figs 1C–D , 2F–J

##### Description.

Length including forewing 4.6 mm. head width across eyes 1.6; pronotum width: 1.3; height of face (crown apex to anteclypeus apex, approximate) 1.0; forewing length 3.4; forewing maximum width (across approximately midlength) 1.2 mm; front tibia length 0.7; hind tibia length 1.7; hind tarsus length 0.7; ovipositor length (portion exposed posterad of sternite VII) 0.9. Hind tibia rows AD, AV and PV with 8, 8 and >17 macrosetae, respectively (PD not visible and PV only partly visible in holotype). Other structural features as described for genus. Dorsal coloration uniformly black except pale distal third of forewing (possibly an artifact of preservation), legs testaceous except for black macrosetal sockets on hind tibia. Female sternite VII only slightly longer than sternite VII, posterior margin shallowly obtusely emarginate.

##### Etymology.

The species name, *obscurus*, refers to the dark overall coloration.

##### Material examined.

Holotype female, Eocene Baltic amber (37–44 Ma), purchased by the first author from an amber dealer in Palanga, Lithuania. Deposited in the Paleontological Collection of the Illinois Natural History Survey (INHSP 10321).

The holotype is well preserved and intact with the right side of the body well visible in dorsal view but the left side largely obscured by a fracture in the amber extended along the midline. In ventral view, much of the head and parts of the legs are obscured by fractures and a milky veil also obscures parts of the legs and abdomen.

**Figure 2.**
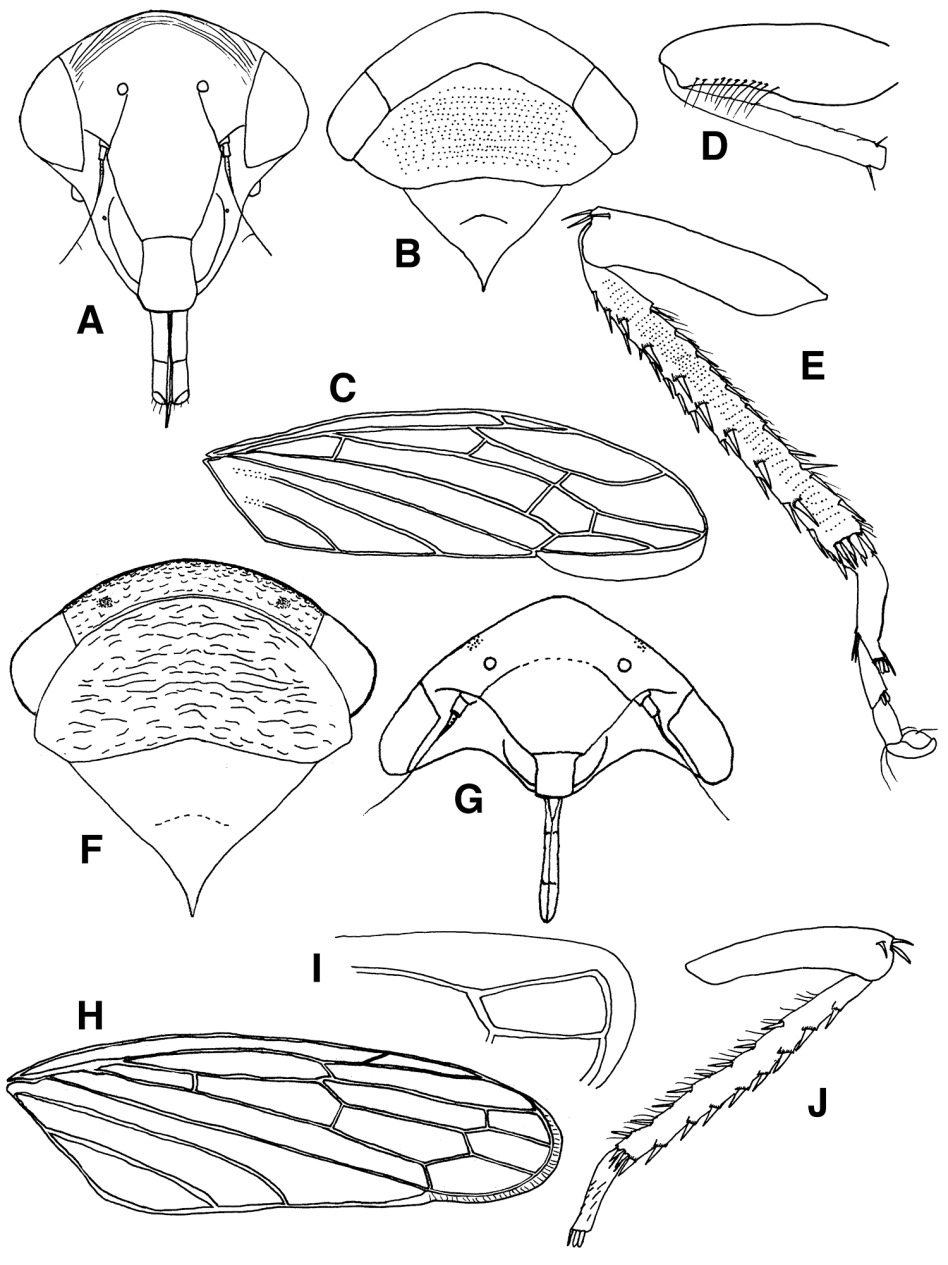
**A–E** *Eoidiocerus emarginatus*: **A** head, anteroventral view **B** head, pronotum and mesonotum, slight anterodorsal view **C** forewing **D** prothoracic femur and tibia, anterior view **E** hind femur, tibia and tarsus, anterior view **F–J** *Archipedionis obscurus* **F** head, pronotum and mesonotum, slight anterodorsal view **G** head, ventral view **H** forewing **I** visible part of hind wing **J** hind femur, tibia and tarsomere, anterior view.

### Subfamily Signoretiinae Baker, 1915

#### Tribe Phlogisini Linnavuori, 1979

##### Priscacutius denticulatus

Taxon classificationAnimaliaHemipteraCicadellidae

Poinar & Brown, 2018, new placement

###### Notes.

This recently described fossil taxon from mid-Cretaceous Myanmar (Burmese) amber (~99 Ma) was originally considered unplaced to subfamily (Poinar and Brown 2018). The holotype fossil was not re-examined but, based on the original photos and description, this species may be confidently placed in the modern subfamily Signoretiinae (new placement) based on the enlarged, punctate pronotum that extends to the scutellar suture (Poinar and Brown 2018: fig. 4). Poinar and Brown (2018) labeled the posterior part of the pronotum as the mesonotum but we interpret the entire sclerite (labeled “P” and “M” in their fig. 4) as the pronotum. *Priscacutius* Poinar & Brown, 2018 runs to tribe Phlogisini in the key of Takiya et al. (2013) based on the position of the ocelli on the crown, distant from the anterior margin and the lack of distinct carinae on the crown and face. On this basis it is here included in Phlogisini although it exhibits several unique features. The tuberculate sensillum adjacent to the antenna, the relatively broad forewing with truncate apex, and the enlarged preapical teeth and elongate apical spines of the first hind tarsomere distinguish *Priscacutius* from previously known genera of Signoretiinae (Takiya et al. 2013, Viraktamath and Dietrich 2017).

## Supplementary Material

XML Treatment for Eurymelinae

XML Treatment for Eoidiocerus

XML Treatment for Eoidiocerus emarginatus

XML Treatment for Archipedionis

XML Treatment for Archipedionis obscurus

XML Treatment for Priscacutius denticulatus
